# Geographical differences of carbapenem non-susceptible *Enterobacterales* and *Acinetobacter* spp. in Germany from 2017 to 2019

**DOI:** 10.1186/s13756-021-01045-z

**Published:** 2022-02-04

**Authors:** Anja von Laer, Tim Eckmanns, Benedikt Zacher, Niels Pfennigwerth, Sören G. Gatermann, Felix Reichert, Michaela Diercke, Gyde Steffen, Doris Altmann, Annicka Reuss

**Affiliations:** 1 Department of Infectious Disease Epidemiology, Robert Koch-Institute, Berlin, Germany; 2 Postgraduate Training for Applied Epidemiology, Robert Koch-Institute, Berlin, Germany; 3 European Programme for Intervention Epidemiology Training, European Centre for Disease Prevention and Control (ECDC), Stockholm, Sweden; 4 German National Reference Centre for Multidrug-Resistant Gram-Negative Bacteria, Department of Medical Microbiology, Ruhr-University Bochum, Bochum, Germany

**Keywords:** Carbapenems, Antibiotic Resistance, Epidemiology, Germany, Public Health, *Enterobacterales*, *Acinetobacter*

## Abstract

**Background:**

Since May 2016, infection and colonisation with carbapenem non-susceptible *Acinetobacter* spp. (CRA) and *Enterobacterales* (CRE) have to be notified to health authorities in Germany. The aim of our study was to assess the epidemiology of CRA and CRE from 2017 to 2019 in Germany, to identify risk groups and to determine geographical differences of CRA and CRE notifications.

**Methods:**

Cases were notified from laboratories to local public health authorities and forwarded to state and national level. Non-susceptibility was defined as intermediate or resistant to ertapenem, imipenem, or meropenem excluding intrinsic bacterial resistance or the detection of a carbapenemase gene. We analysed CRA and CRE notifications from 2017, 2018 and 2019 per 100,000 inhabitants (notification incidence), regarding their demographic, clinical and laboratory information. The effect of regional hospital-density on CRA and CRE notification incidence was estimated using negative binomial regression.

**Results:**

From 2017 to 2019, 2278 CRA and 12,282 CRE cases were notified in Germany. CRA and CRE cases did not differ regarding demographic and clinical information, e.g. proportion infected. The notification incidence of CRA declined slightly from 0.95 in 2017 to 0.86 in 2019, whereas CRE increased from 4.23 in 2017 to 5.72 in 2019. The highest CRA and CRE notification incidences were found in the age groups above 70 years. Infants below 1 year showed a high CRE notification incidence, too. Notification incidences varied between 0.10 and 2.86 for CRA and between 1.49 and 9.99 for CRE by federal state. The notification incidence of CRA and CRE cases increased with each additional hospital per district.

**Conclusion:**

The notification incidence of CRA and CRE varied geographically and was correlated with the number of hospitals.The results support the assumption that hospitals are the main driver for higher CRE and CRA incidence. Preventive strategies and early control measures should target older age groups and newborns and areas with a high incidence.

**Supplementary Information:**

The online version contains supplementary material available at 10.1186/s13756-021-01045-z.

## Background

Gram-negative bacteria that are non-susceptible to carbapenems pose a significant threat to patients and healthcare systems and are a major public health issue [1]. In the priority pathogens list of the World Health Organization (WHO) carbapenem-resistant *Acinetobacter* spp. and *Enterobacterales* are classified as the most critical group of bacteria that pose the greatest threat to human health [2].

Whereas *Acinetobacter* spp. is mainly disseminated via environmental contamination, *Enterobacterales* are mainly transmitted person-to-person, i.e. via the hands of health care workers [3]. These bacteria occur especially in hospital settings due to antibiotic use and co-morbidities of the patients, but new studies suggest that also non-healthcare associated transmission outside health care settings occur [4–6]. Identifying risk groups for carriage of carbapenem non-susceptible *Acinetobacter* spp. (CRA) and *Enterobacterales* (CRE) might guide local public health authorities to implement preventive strategies to prevent further spread. Identifying differences in geographical distribution of CRA and CRE helps to adapt control measures to local situations.

Carbapenem non-susceptibility varies between countries within the European Union and between bacterial species [1, 7]. In many European countries, these resistant bacteria have increased since 2012 and especially high levels are reported for *Klebsiella pneumoniae* and *Acinetobacter* spp. [7–9].

Until 2016, surveillance data on antimicrobial resistance in Germany were only available from the German Antimicrobial Resistance Surveillance (ARS) System which collects routine data of antimicrobial susceptibility testing from voluntarily participating German laboratories [10]. Based on data from ARS, carbapenem non-susceptibility in *K. pneumoniae* was present in 0.5% of all clinical isolates in 2016 [11]. The percentage of invasive carbapenem resistant *Acinetobacter* spp. was 4.5% in 2018 [7]. Although Germany has a low proportion of carbapenem resistant bacteria, it has been slowly increasing over the past years [11].

In 2016, a new comprehensive surveillance for CRA and CRE was implemented in Germany to assess the epidemiological situation based on nationwide data [12, 13]. The mandatory notification requirements include infection as well as colonisation. These surveillance data focus primarily on the epidemiology and do not aim to identify what was driving the carbapenem non-susceptibility (i.e. causing genes, clonal or plasmid mediated dissemination). The early identification and notification of cases to local public health authorities (LPHA) aims to facilitate rapid implementation of control measures to avoid further spread.

The aim of our study was to assess the epidemiology of CRA and CRE and to identify risk groups for carriage of CRA and CRE. A special focus was laid on geographical differences from 2017 to 2019 in Germany to support public health authorities to target infection control measures locally and nationally and to determine the influence of hospitals on CRA and CRE notifications.

## Methods

### Data and data sources

#### Notification data

We analysed CRA and CRE notifications from 2017, 2018 and 2019. Data were extracted from the German national surveillance system data base SurvNet@RKI as of 01 March 2020 [14, 15]. Pseudonymized case-based notification data are available at national level. Collected data included demographic, clinical and laboratory information, i.e. age, sex, federal state, notification date, infection/colonisation, hospitalisation, sample material, bacterial species, carbapenemases and outbreak information.

#### Hospital data

Data on hospitals in Germany were available from the directory and the basic data of hospitals published by the German Federal Statistical Office from 2017 [16, 17].

### Case definition

Cases were notified from laboratories to LPHA and electronically forwarded to state and national public health authorities (Robert Koch Institute, RKI). Cases were forwarded to state health authorities usually by the LPHA responsible for the permanent address of the patient.

At LPHA level, CRE or CRA cases were categorised according to the case definition issued by RKI [18]. The case definition required a laboratory confirmed infection or colonisation with *Acinetobacter* spp. or *Enterobacterales* by culture or PCR with non-susceptibility to at least one carbapenem. Non-susceptibility was defined as an intermediate or resistant phenotype to ertapenem, imipenem, or meropenem in antimicrobial susceptibility testing or detection of a carbapenemase gene. Intrinsic bacterial resistance towards carbapenems was disregarded. Intrinsic non-susceptiblity to ertapenem was assumed in *Acinetobacter* spp., *Enterobacter* spp., *Citrobacter* spp. and *Klebsiella aerogenes*, intrinsic non-susceptibility to imipenem was assumed in *Morganellaceae* and *Serratia marcescens *[19, 20]. *Enterobacter* spp., *Citrobacter* spp. and *Klebsiella aerogenes* are often non-suscepible to ertapenem. In *Enterobacterales* non-susceptibility to ertapenem is often caused by other mechanisms than carbapenemases and overexpression of AmpC-β-lactamases results in non-susceptible isolates [21, 22]. As the EUCAST (European Committee on Antimicrobial Susceptibility Testing) changes in interpretation of susceptibility categories did not affect the screening practices for carbapenemases, this did not affect the notification of CRA and CRE.

If CRA or CRE were repeatedly identified in the same patient, a new notification had to be forwarded for every admission to hospital or after three months for outpatients. Colonisation or infection with multiple CRA or CRE should result in multiple notifications. Thus, one case does not necessarily represent one patient.

Case definition for CRA and CRE according to RKI [23]:

A case has to be forwarded to RKI if it fulfils the following requirements:

- Direct detection of the bacteria with
  o Culture or nucleic acid detection
- AND
- Detection of carbapenem non-susceptibility or detection of a carbapenemase with
  o Antimicrobial susceptibility testing or detection of a carbapenemase gene

### Analyses

The completeness of data was determineded by the proportion of missing information for administrative, demographic, clinical and laboratory details.

To assess the epidemiological situation, cases of CRE and CRA were described by their demographic, clinical and laboratory characteristics over time. The notification incidence (the number of notified cases per 100,000 inhabitants) per age group (< 1, 1–9, 10–19, 20–29, 30–39, 40–49, 50–59, 60–69, 70–79, 80 + years), sex and federal state was calculated.

To test the hypothesis that areas with a high hospital density have a higher notification incidence, we compared the notification incidence of hospitalised CRE and CRA cases with the number of hospitals and hospital beds in each district using negative binomial regression accounting for population. A district is the administrative unit with responsibility of one LPHA in Germany. Hospitals were divided in general hospitals (non-university hospitals) and university hospitals as classified in the directory of hospitals in Germany. The analysis was repeated with all notified cases irrespective of hospitalisation status. We calculated the number of notified hospitalised cases and the number of cases per 10,000 treated inpatients and per 1000 patient-days for each federal state. A *p*-value (*p*) below 0.05 was defined as statistically significant.

Data analysis was performed using Stata® version 15.1 (Stata Corp, Texas, USA) and Microsoft Excel 2010 (Microsoft, Washington, USA). Graphical illustrations were created using Regiograph Analyse (GfK, Nurnberg, Germany).

### Data protection and ethics

CRA and CRE notification data were collected within the legal framework of the Protection against Infection Act. In this paper only aggregated data are shown. Therefore, no data protection declaration or ethics committee vote was necessary.

## Results

**Fig. 1:**
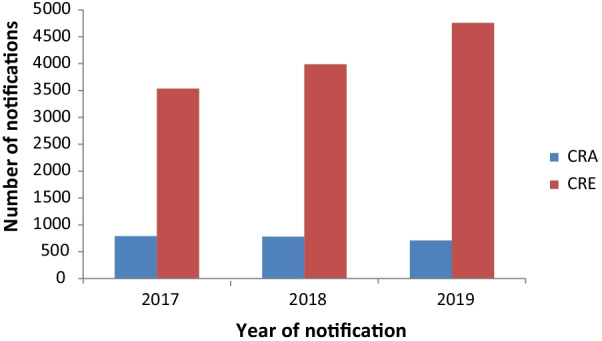
Number of CRA (n = 2278) and CRE (n = 12,282) notifications, Germany, 2017–2019

In total, 2278 carbapenem non-susceptible *Acinetobacter* spp. (CRA) and 12,282 carbapenem non-susceptible *Enterobacterales* (CRE) were notified in Germany from 2017 to 2019 (Fig. 1). The number of CRA notifications was stable over this time period, whereas the number of CRE notifications increased from 2017 to 2019. Of 2102 CRA and 11,100 CRE with information, carbapenem non-susceptibility was determined primarily with culture-based antimicrobial susceptibility testing in 97.9% (n = 2057 and n = 10,869, respectively).

### Data completeness

Completeness of data was similar in CRA and CRE cases and varied between 100.0 and 57.3% over the three-years period depending on notified variables. Information on demographics had the highest proportion of completeness (100.0%). Clinical information, e.g. if a patient was colonised or infected (69.7%), and data on microbiological results, e.g. if a test for carbapenemases was performed (57.3%), was reported less often (Additional file 1).

### Epidemiology of CRA and CRE

#### Demographics

CRA and CRE cases had a similar median age of 66 years and 68 years, respectively (Table 1). More men than women were notified with CRA (66%) and CRE (62%). These demographics did not change in the study period.

**Table 1:** Demographic, clinical and laboratory information of notified CRA (n = 2278) and CRE (n = 12,282) cases, Germany, 2017–2019

Characteristic	Cases	2017		2018		2019		2017–2019	
		n/N	%	n/N	%	n/N	%	n/N	%
Male sex	CRA	514/786	65.4%	517/780	66.3%	464/710	65.4%	1495/2276	65.7%
	CRE	2186/3522	62.1%	2424/3966	61.1%	2958/4742	62.4%	7568/12,230	61.9%
Median age in years (IQR)	CRA	66	(53;75)	66	(51;76)	67	(54;77)	66	(53;76)
	CRE	67	(52;77)	68	(55;78)	68	(55;77)	68	(54;77)
Hospitalisation	CRA	664/742	89.5%	646/730	88.5%	595/660	90.2%	1905/2132	89.4%
	CRE	3030/3332	90.9%	3380/3735	90.5%	4023/4462	90.2%	10,433/11,529	90.5%
Infection	CRA	193/499	38.7%	196/607	32.3%	179/523	34.2%	568/1629	34.9%
	CRE	744/2129	34.9%	938/2939	31.9%	1113/3450	32.3%	2795/8518	32.8%
Carbapenemase test performed	CRA	226/331	68.3%	353/415	85.1%	353/410	86.1%	932/1156	80.6%
	CRE	1193/1505	79.3%	1756/2101	83.6%	2410/2796	86.2%	5359/6402	83.7%
Carbapenemase detected	CRA	201/226	88.9%	316/353	89.5%	310/353	87.8%	827/932	88.7%
	CRE	925/1193	77.5%	1290/1756	73.5%	1813/2410	75.2%	4028/5359	75.2%
Part of an outbreak	CRA	62/787	7.9%	38/780	4.9%	37/711	5.2%	137/2278	6.0%
	CRE	85/3440	2.5%	88/3934	2.2%	104/4670	2.2%	277/12,044	2.3%

**Fig. 2:**
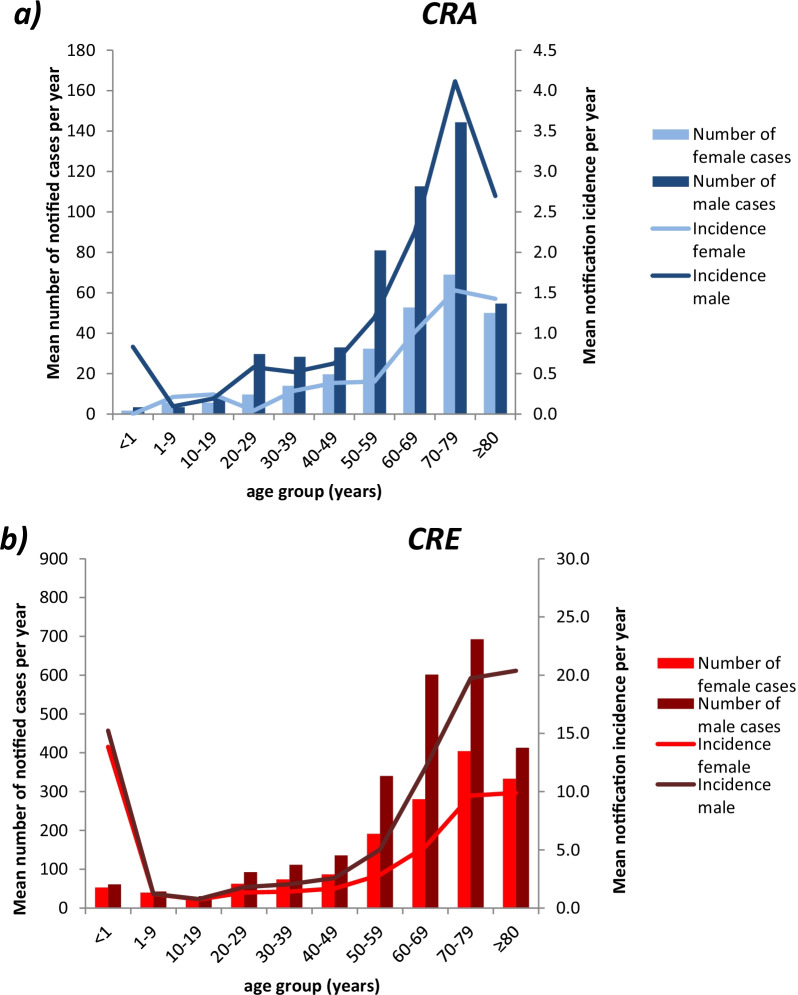
**a** Number of CRA (n = 2275) and **b** Number of CRE (n = 12,217) notifications and notification incidences by sex and age group, Germany, 2017–2019

Most CRA and CRE cases were observed in the age groups from 50 years onwards (Fig. 2). For both males and females, the highest notification incidences were found in the age group of 70 to 79 years and over 80 years in CRA and CRE, respectively. Additionally, among CRE cases, infants below 1 year of age showed a high notification incidence, but the number of cases was low in this age group. In most age groups, males were more often affected than females. In 2019, three CRE cases were notified as diverse gender in the age groups < 1 year, 20–29 years and 70–79 years, respectively.

### Clinical and laboratory information

The proportion of hospitalised CRA and CRE cases was similar (90% and 91%, respectively) (Table 1). Infants below one year and cases aged 50 years and above had a higher proportion of hospitalisation (95% and 91%) than other age groups (75–88%). In total, 35% of CRA and 33% of CRE cases were notified as being infected with the respective bacterium. Of all notified cases, 34 CRA cases (1.49%) and 83 CRE cases (0.68%) the infection resulted in the death of patients according to the reported data. The proportion of cases belonging to an outbreak was higher in CRA (5–8%) than in CRE (2–3%). Most notified CRE outbreaks were caused by *Klebsiella pneumoniae*.

### Bacterial species

**Fig. 3:**
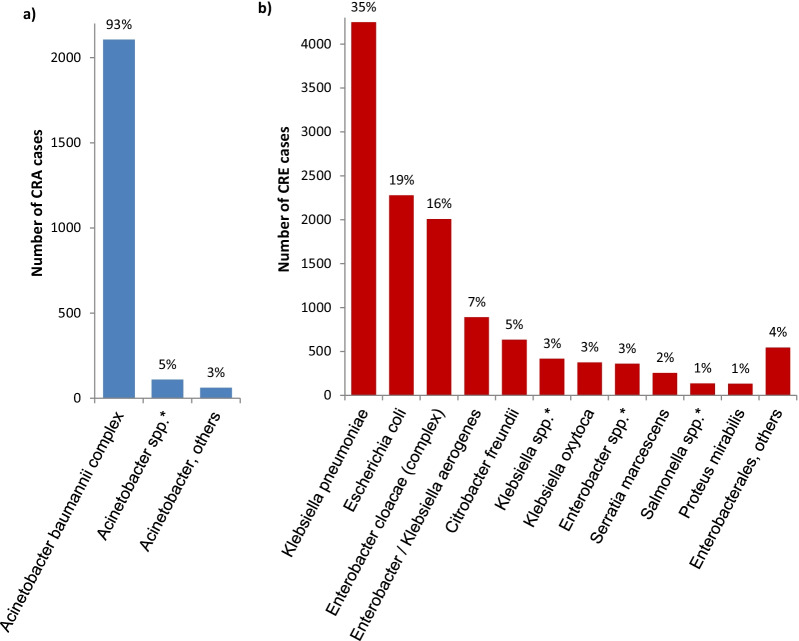
Most common reported bacteria in **a** notified CRA cases (n = 2278) and **b** notified CRE cases (n = 12,282), Germany, 2017–2019. *Certain notifications included bacteria on genus level only, the species was not specified in these cases

In CRA cases, the most common notified species belonged to *Acinetobacter baumannii*-complex (93%), followed by *Acinetobacter* spp. without differentiation to species level (5%) (Fig. 3). In CRE cases, *Klebsiella pneumoniae* was reported in 35% of cases, followed by *Escherichia coli* (19%) and *Enterobacter cloacae*-complex (16%). These distributions did not change over the study period (Additional file 2).

CRE were more often detected in screening samples than CRA (48% and 39%, respectively). If detected in clinical samples, CRA were reported in wound swabs in 40% followed by respiratory material in 33% of cases; whereas CRE were reported in urine in 42% followed by wound swabs in 23% of cases. In the group of carbapenem non-susceptible “*Enterobacterales,* other”, 23 *Yersinia* spp. and 10 *Shigella* spp. were notified.

### Carbapenemases

**Fig. 4:**
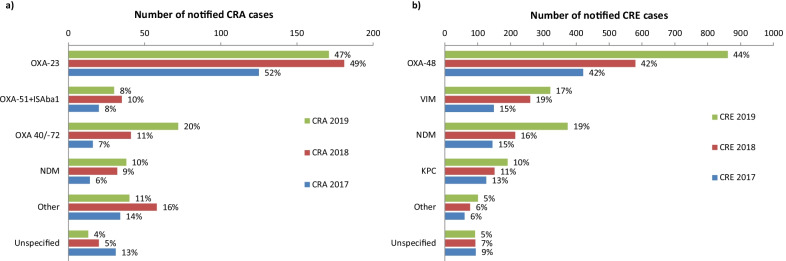
Number and proportion of detected carbapenemases in **a** notified CRA cases (n = 1114) and **b** notified CRE cases (n = 4296), multiple entries possible per case, Germany, 2017–2019

From 2017 to 2019, it was specified whether a test for carbapenemase genes was performed in the laboratory on average for 51% of CRA and for 52% of CRE cases with an increasing trend over the years. If a test was performed, a carbapenemase was detected in 89% of CRA cases and 75% of CRE cases. The most common detected carbapenemases in CRA were OXA-23, OXA-51 (IS*Aba1* upstream of *bla*_OXA-51-like_) and OXA-40/-72 (Fig. 4). The most common detected carbapenemases in CRE were OXA-48-like, VIM and NDM.

### Geographical distribution

**Fig. 5:**
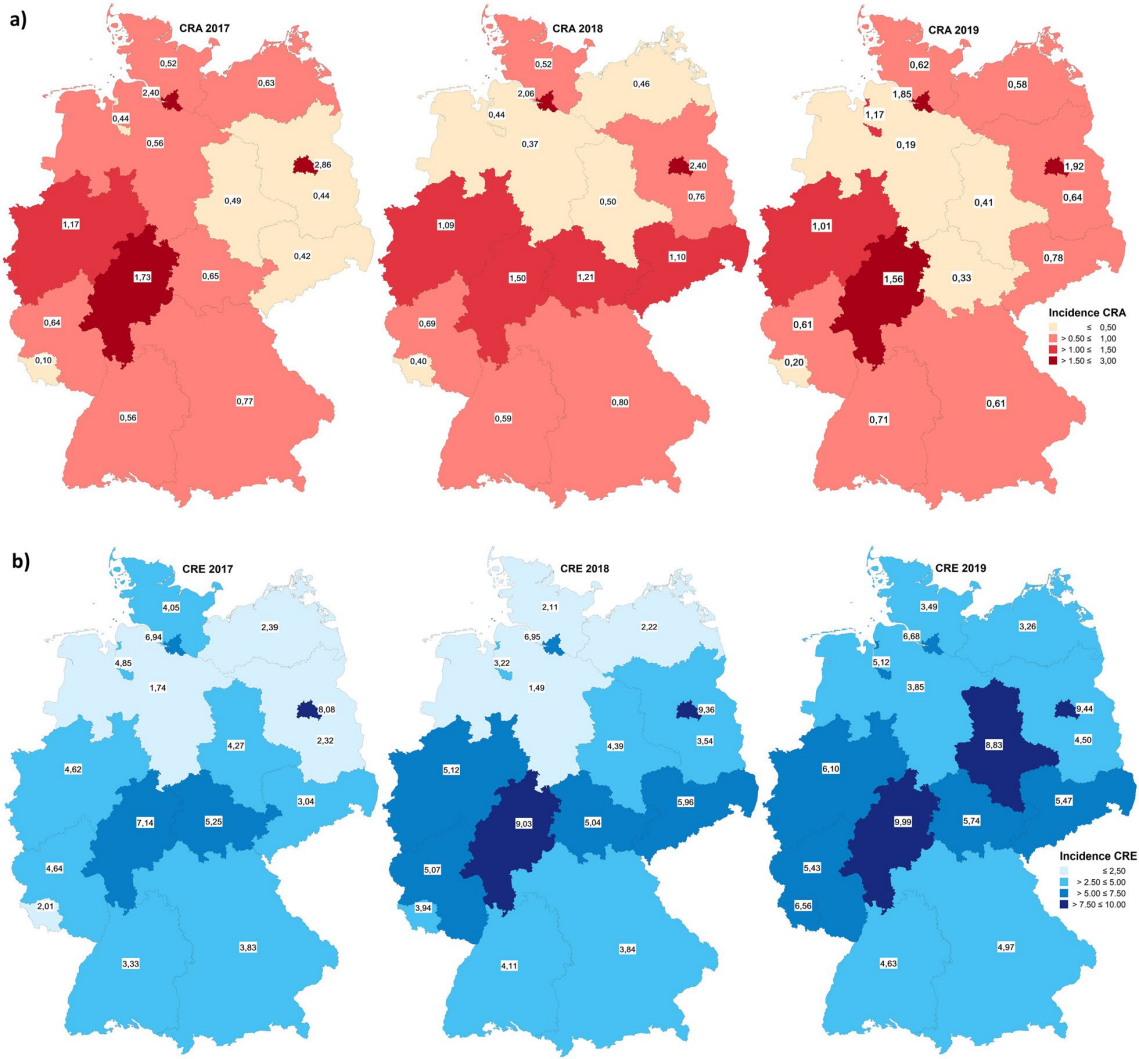
Notification incidences of **a** CRA (n = 2278) and **b** CRE (n = 12,279), by federal state, Germany, 2017–2019

The notification incidence of CRA and CRE differed by federal state (Fig. 5). The overall notification incidence of CRA declined slightly in the study period with 0.95 (2017), 0.94 (2018) and 0.86 (2019) notifications per 100,000 inhabitants. The lowest CRA incidence was notified from Saarland (0.10 in 2017) and the highest from Berlin (2.86 in 2017). The overall incidence of CRE increased from 2017 to 2019 with 4.23 (2017), 4.80 (2018) and 5.72 (2019) notifications per 100,000 inhabitants. The lowest CRE incidence was notified from Lower Saxony (1.49 in 2018) and the highest from Hesse (9.99 in 2019).﻿﻿﻿

### Correlation between notification incidence and health care structure

The number of hospitals ranged from 0 to 79 hospitals per district (Additional file 3).

The number of notified hospitalised CRA cases was 0.33–0.36 per 10,000 inpatients in Germany. This varied between federal states from 0.00–0.95 cases/10,000 inpatients. From 2017 to 2019, 0.01 hospitalised CRA cases per 1000 patient-days were notified each year. This varied from 0.00–0.02 cases/1000 patient-days per federal state. The number of hospitalised CRE cases per 10,000 inpatients increased over the years from 1.66 in 2017 to 1.87 in 2018 and 2.22 in 2019. This varied between federal states from 0.31 to 4.28 cases/10,000 inpatients. In CRE, 0.02 cases per 1000 patient-days were notified in 2017, 0.03 in 2018 and 2019. This varied from 0.00–0.06 cases/1000 patient-days per federal state.

Districts with university hospitals had a higher notification incidence of hospitalised CRA [Incidence Rate Ratio (IRR): 1.74, 95% Confidence Interval (CI): 1.29–2.34, *p* < 0.01] and CRE cases [IRR: 1.71, 95% CI: 1.33–2.19, *p* < 0.01] than districts without university hospitals. In the univariable analysis the notification incidence of CRA and CRE cases was significantly positively correlated with the number of general hospitals, university hospitals and hospital beds per district (Additional file 4). In the multivariable analysis number of hospitals and hospital beds were positively correlated with the notification incidence of CRA and CRE, but not all were statistically significant (Table 2). CRA incidence increased by 2–3% with each additional hospital and by 24–47% with each additional university hospital. CRE incidence increased by 0.5–0.8% with each additional hospital and by 40–64% with each additional university hospital. The repeated analyses including all notified cases irrespective of hospitalisation status revealed similar results (data not shown).

**Table 2:** Multivariable analysis of type of hospital, hospital density and hospital size and notification incidence of notified hospitalised CRA (n = 2051) and CRE (n = 11,183) per district, Germany, 2017–2019

Variable	Notified cases	2017			2018			2019		
		IRR^1^	[95% CI^2^]	*p*-value	IRR^1^	[95% CI^2^]	*p*-value	IRR^1^	[95% CI^2^]	*p*-value
Hospitals	CRA	1.030200	[1.009963–1.050842]	< 0.01	1.015363	[0.998942–1.032055]	0.07	1.019253	[1.002559–1.036226]	< 0.05
	CRE	1.007100	[0.994128–1.020241]	0.29	1.008303	[0.995344–1.021430]	0.21	1.005281	[0.992819–1.017899]	0.41
University hospitals	CRA	1.237825	[0.853176–1.795890]	0.26	1.471903	[1.022384–2.119064]	< 0.05	1.368048	[0.967789–1.933846]	0.08
	CRE	1.454999	[1.097646–1.928691]	< 0.05	1.401988	[1.058177–1.857505]	< 0.05	1.641121	[1.269523–2.121487]	< 0.01
Hospital beds	CRA	1.000168	[1.000076–1.000260]	< 0.01	1.000090	[1.000004–1.000176]	< 0.05	1.000113	[1.000033–1.000194]	< 0.01
	CRE	1.000040	[0.999976–1.000105]	0.22	1.000049	[0.999984–1.000113]	0.14	1.000039	[0.999977–1.000102]	0.22
University hospital beds	CRA	1.000047	[0.999773–1.000321]	0.74	1.000202	[0.999933–1.000471]	0.14	1.000126	[0.999873–1.000379]	0.33
	CRE	1.000247	[1.000048–1.000451]	< 0.05	1.000231	[1.000033–1.000428]	< 0.05	1.000310	[1.000124–1.000496]	< 0.01

^1^IRR: Incidence Rate Ratio

^2^95% CI: 95% Confidence Interval

## Discussion

We described the epidemiology of carbapenem non-susceptible *Acinetobacter* spp. (CRA) and *Enterobacterales* (CRE) that were notified in Germany from 2017 to 2019.

The notification incidence was 0.95, 0.94 and 0.86 for CRA and 4.23, 4.80 and 5.72 for CRE in 2017, 2018 and 2019, respectively. Whereas the notification incidence of CRA decreased over the study period, the notification incidence of CRE increased by 35%. This is in accordance with the finding that the proportion of carbapenem non-susceptible *K. pneumoniae* increased in Germany in the last years [11]. Comparison with data from other states is difficult as surveillance systems, test strategies, access to healthcare and clinical routine differ substantially. A study in the United States found an annual notification incidence rate of CRE infections of 2.93 per 100,000 population in 2012, but included certain infections only and did not include colonisations [24].

Median age of notified CRA cases was 66 years and of CRE cases 68 years. The age and gender structure of notified cases reflect that of hospital patients in Germany [25]. High CRA and CRE incidences were found in patients over 70 years and for CRE in infants under one year of age. Among the older age groups, men were more affected. This is in accordance with the literature and emphasizes that hospitalisation might be the main driver of CRA and CRE [26]. The notification incidence of CRE in infants below one year of age might also be elevated because of the recommended screening of newborns for certain bacteria in neonatal intensive care [27]. This is supported by the finding that 86% of cases below one year of age were reported to be colonised. In all age groups, most cases were colonised with CRA or CRE, but about a third of cases had an infection. Death due to the notified illness should be interpreted with caution as it is unknown if and how LPHA follow-up on cases and underreporting with huge regional variation is assumed.

The most common reported bacteria were *Klebsiella pneumoniae* and *Acinetobacter baumannii-*complex. The most common reported carbapenemase in CRE was OXA-48 and in CRA OXA-23. Both results are in accordance with the findings of the National Reference Centre for Multidrug-resistant Gram-negative bacteria (NRC) in Germany [28]. The NRC receives isolates sent voluntarily from diagnostic laboratories to confirm carbapenem non-susceptibility.

The notification incidence of CRA and CRE varied geographically with the highest notification incidence in the federal states of Berlin, Hamburg and Hesse. The geographical variation could be due to different screening practices in health care facilities, a better reporting compliance in these federal states or because of a real higher occurrence of these bacteria. German hospitals are encouraged to standardise their screening practices according to the recommendations of the Commission for Hospital Hygiene and Infection Prevention (KRINKO), but screening practices might differ between hospitals [29]. Enhanced surveillance due to active surveillance in outbreak situations could also increase the incidence. Hamburg and Berlin are two city states with big university hospitals. Berlin published outbreaks with extended-spectrum β-lactamase producing *Enterobacterales* and CRE in 2016 [30, 31]. Hesse had implemented a mandatory notification for carbapenem non-susceptible Gram-negative bacteria already in 2011, therefore it seems possible that reporting compliance might be better established than in other federal states [32]. Within the notification data, the district (unit of one responsible LPHA) is mainly allocated according to the patient’s permanent address and does not necessarily represent the place of exposure. A local study showed that almost 60% of cases notified from the laboratory to the LPHA in the district of Frankfurt (Hesse) were not residents in Frankfurt [33]. These cases might have been forwarded to national level by the LPHA responsible for the district of residence of the patient and would therefore be allocated to another district than the one where the exposure occurred. We did not include place of exposure in our analysis, as only less than 1% of cases (n = 95/14,560) reported place of exposure.

Mean yearly notification incidence per 1000 patient-days was 0.01 for CRA and 0.03 for CRE. In international studies the notification incidence of carbapenem non-susceptible Gram-negative bacteria varied from 0.007 to 35.2 per 1000 patient-days, but these studies included *Pseudomonas aeruginosa* and high risk areas only, but often did not include colonisation [34–37].

As transmission of CRA and CRE occurs mostly within healthcare settings, one would assume that the number of notifications is highest in areas with a high hospital density and therefore more hospitalised patients [4]. We could support this finding; hospitals and hospital beds increased the notification incidence of CRA and CRE. This is in line with our finding that more often hospitalised age groups show a higher notification incidence. Whereas the notification incidence of CRA seems to be linked to the number of hospitals and hospital beds more generally, the incidence of CRE seems to be linked to university hospitals. This is in accordance with an analysis of carbapenem non-susceptible *Klebsiella pneumoniae* in Germany that identified highly specialised hospitals and intensive care units as risk factors [11]. The association between hospital type and the incidence of CRA notifications was not significant for year-wise but for cumulative analyses. Therefore, the small sample size in university hospitals and CRA cases might have led to a false non-significant result. Additionally, intensified infection prevention measures and antimicrobial stewardship programs in university hospitals impact the transmission within the hospital and might decrease the notification incidence. Nonetheless, the impact of hospitals might be underestimated because place of exposure could not be analysed. This could impact especially university hospitals as those specialised units have large catchment areas and cases might be allocated to their district of residence. Complicated and difficult to treat cases are often referred to highly specialized and university hospitals. These cases often have a prolonged healthcare journey and were more exposed to healthcare settings. Furthermore, specialized and university hospitals might have a more intensive screening strategy in place. The directory of hospitals includes every hospital in Germany irrespective of number of beds. This finding might also lead to an underestimation of the association of the notification incidence and general hospitals. Geographical differences in health care seeking behaviour might impact the number of hospitalised patients and the notification incidence of CRA and CRE, although a significant association between the social deprivation index and the proportion of carbapenem non-susceptible *Klebsiella pneumoniae* in Germany could not be shown [11]. Hospitalisations in Germany differ regionally due to demographic and social structure, urbanisation, population density, and health status and supply of hospital beds impacts hospital utilisation (supply-sensitive care) [38]. This should be investigated further. It is known that CRE are also spread in the community, but data on community-acquired CRE are scarce [39, 40]. As health care structure also reflects social structure and population density, this may especially influence the notification incidence of CRE. As university hospitals might have intensified surveillance programs, the strong association of the number of university hospitals and the notification incidence of CRE might reflect a much higher incidence of CRE within the community. The national recommendation which risk groups should be screened in hospital does not differ for CRA and CRE. Bias due to systematic differences in targeted risk groups of screening for these two bacterial groups is therefore not assumed. Nonetheless, screening practices and the association between highly specialised clinics and the notification incidence of CRA and CRE should be investigated further on hospital level. The analysis should be repeated with notification data from the following years to decide whether the geographical differences of CRE and CRA cases remain stable. Whether other highly specialised hospitals show a similar association as university hospitals, could not be analysed with the available data.

There are several limitations to this study. Completeness of information in the surveillance system is crucial to verify, analyse and interpret notification data. The initial notification from laboratories to LPHA contains usually demographic as well as laboratory information. Additional data, such as clinical information, have to be investigated by the LPHA and the results of specific laboratory tests, such as the test for carbapenemases, is often added to the initial notification at a later time. Although most variables were 75% or more complete, there were missing values in particular with regards to infection status and test for carbapenemases. This additional information was often incomplete. This limits the generalisability of these study results. LPHA as well as hospital and laboratory staff members are encouraged to share information in a timely manner and work closely together. The information investigated by the LPHA should be entered into the notification software and forwarded to national level. Several notifications might have been forwarded for one patient so one case does not necessarily represent one patient. The number of CRA and CRE cases per inpatients and patient-days as well as local risk factors should be investigated and monitored on hospital level. This will be important to assess and support local preventive control measures.

## Conclusion

This is the first report on CRA and CRE notifications in Germany since mandatory notification was established. The notification incidence of CRA and CRE varied geographically and was correlated with the type and number of hospitals and hospital beds per region.The results support the hospital as main driver of CRE and CRA. Whereas CRA seems to be correlated more generally with hospital density, CRE seems especially to be impacted by university hospitals. Both vulnerable groups, the elderly and the infants below one year of age, should be especially targeted when implementing infection control measures. In areas with a high incidence targeted preventive strategies and early control measures should be reviewed and adapted to prevent further spread. Geographical differences should be investigated further to exclude variation in testing and/or notification procedures. Furthermore, the impact of underestimation of cases should be investigated. We also encourage molecular surveillance of CRA and CRE to better understand and prevent transmission.

## Supplementary Information

**Additional file 1.** Completeness of CRA (n = 2,278) and CRE (n = 12,282) notifications, Germany, 2017-2019.

**Additional file 2.** Most common reported bacteria in notified CRA (n = 2,278) and CRE (n = 12,282) cases, Germany, 2017-2019.

**Additional file 3.** Hospitals and university hospitals in Germany per district, 2017. Dots are randomly placed within one district. * According to the directory of hospitals, there were 1,776 general hospitals and 35 university hospitals in Germany in 2017 with a total of 479,893 general hospital beds and 45,156 university hospital beds. A total of 19,442,810 patients were treated in hospitals what accounted for 141,152*1,000 patient-days and patients stayed in hospital for a mean of 7.3 days (range: 6.9-7.9 days by federal state).

**Additional file 4.** Univariable analysis of type of hospital, hospital density and hospital size and notification incidence of notified hospitalised CRA (n = 2,051) and CRE (n = 11,183) per district, Germany, 2017.

## Data Availability

The aggregated datasets analysed during the current study are available online [16, 17]. The detailed datasets of surveillance data analysed during the current study are not publically available due to confidentiality, but are available from the corresponding author on reasonable request.

## Abbreviations

CRE: Carbapenem non-susceptible (resistant or intermediate) *Enterobacterales*
CRA: Carbapenem non-susceptible *Acinetobacter* spp.
WHO: World Health Organization
ECDC: European Centre for Disease Prevention and Control
ARS: German Antimicrobial Resistance Surveillance
RKI: Robert Koch Institute
EUCAST: European Committee on Antimicrobial Susceptibility Testing
LPHA: Local Public Health Authority
KRINKO: Commission for Hospital Hygiene and Infection Prevention
