# Supplementary figures and images for: Geographical differences of carbapenem non-susceptible Enterobacterales and Acinetobacter spp. in Germany from 2017 to 2019
Source: Antimicrob Resist Infect Control. 2022 Feb 4;11:25. doi: 10.1186/s13756-021-01045-z (PMC8815152; doi:10.1186/s13756-021-01045-z)

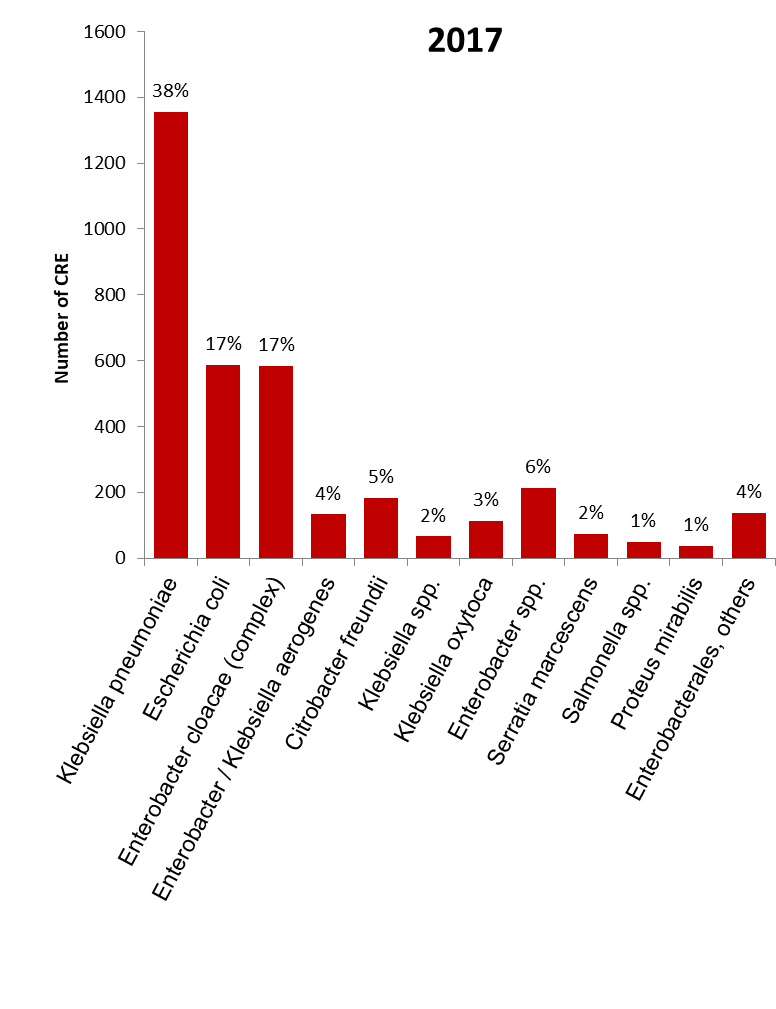

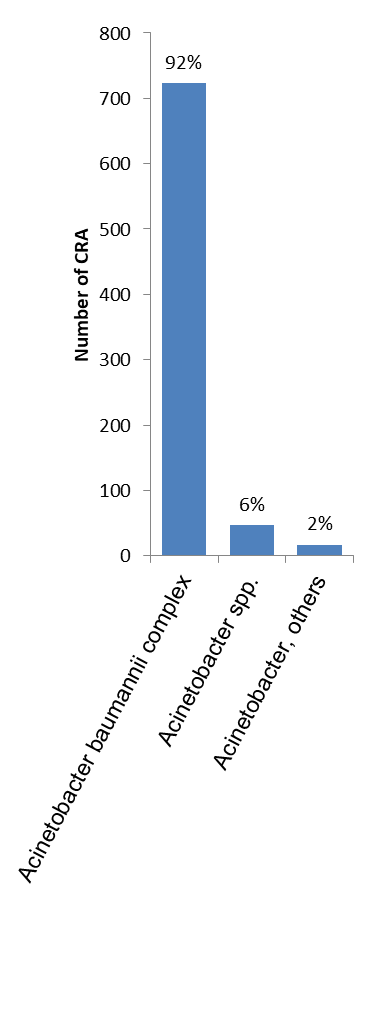


**a)**

**b)**


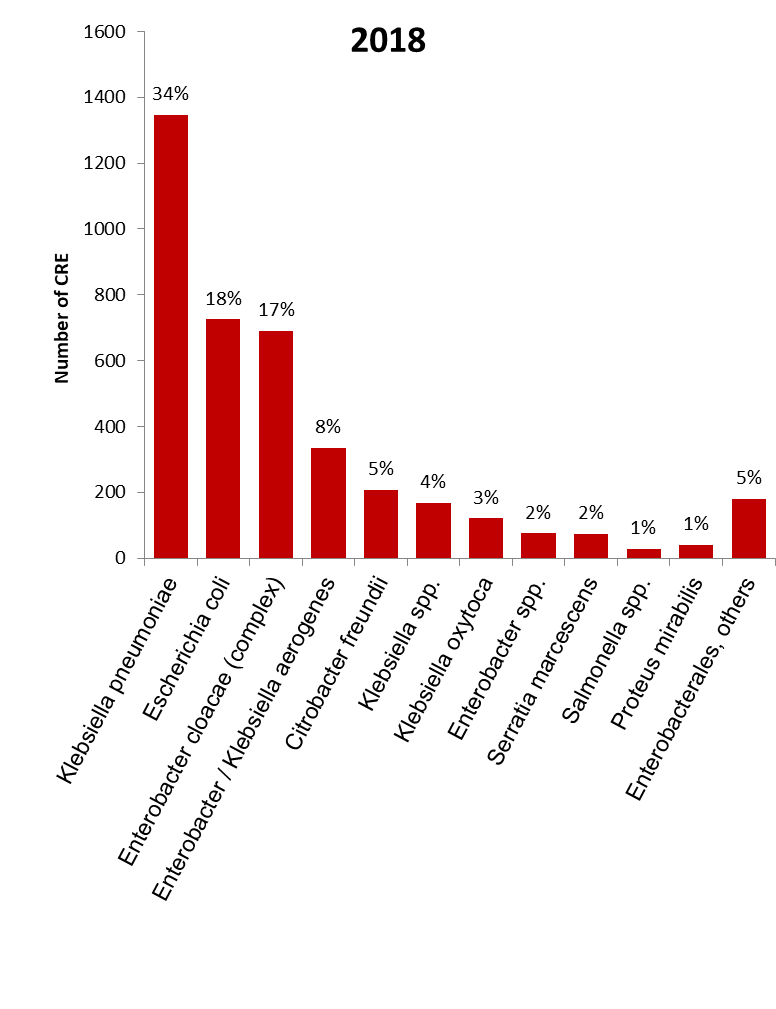

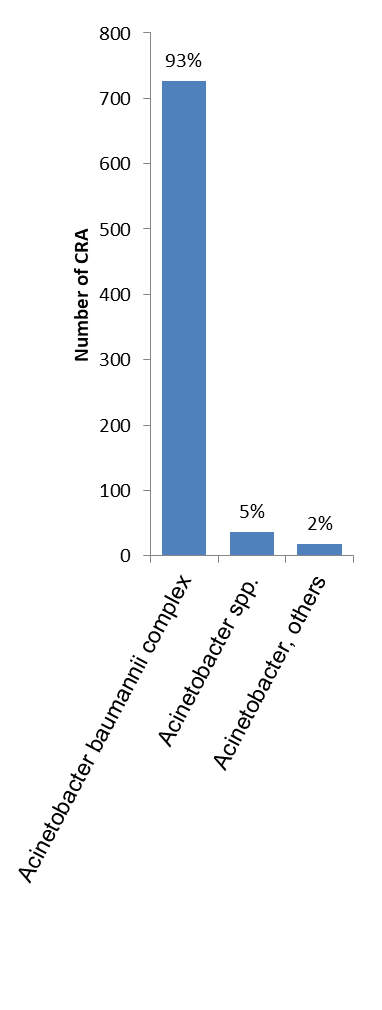


**a)**

**b)**


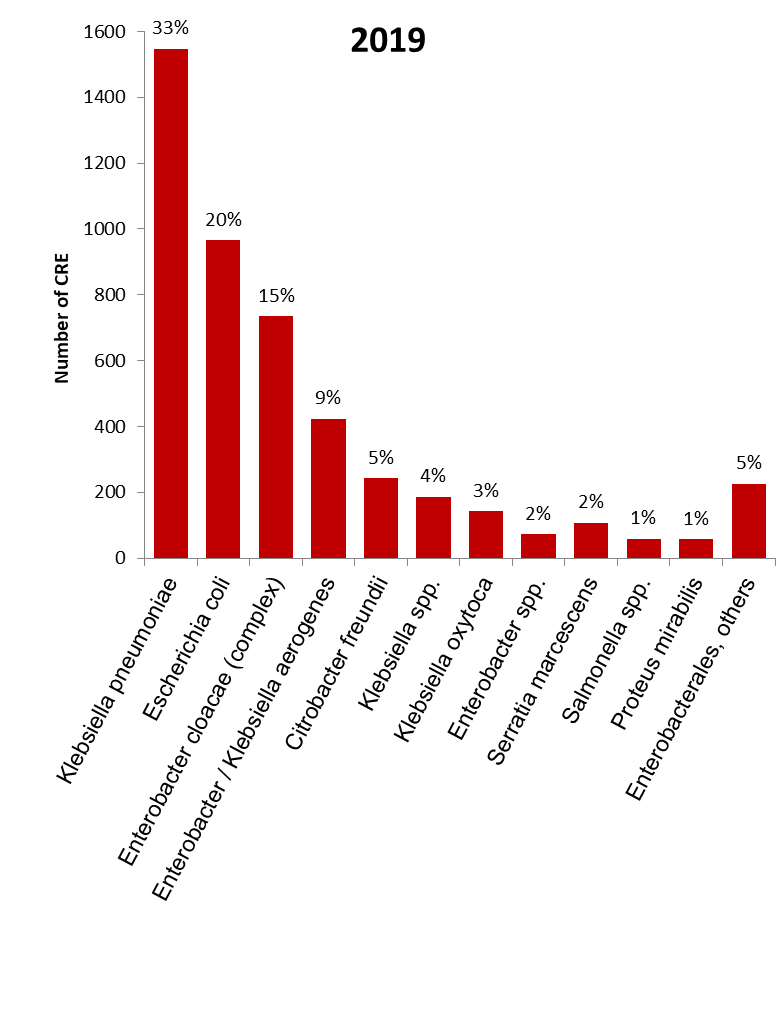

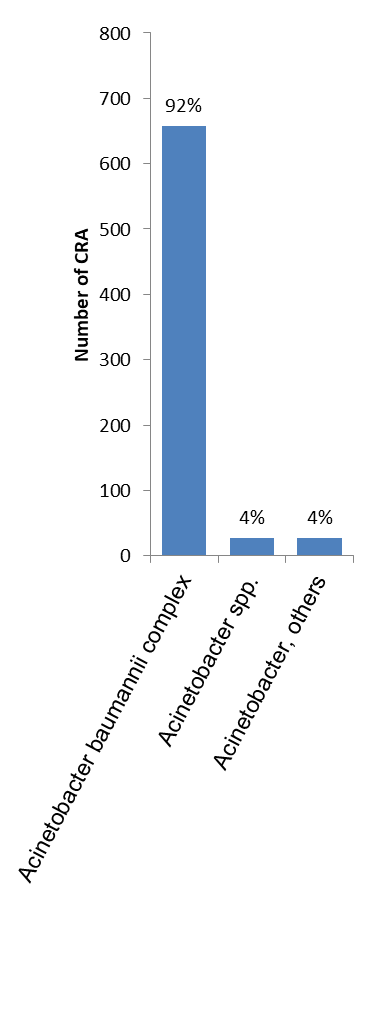


**a)**

**b)**

Supplement: Supplementary file 2 — Additional file 2. Most common reported bacteria in notified CRA (n = 2,278) and CRE (n = 12,282) cases, Germany, 2017-2019. [file 13756_2021_1045_MOESM2_ESM.docx]

## Slide 1
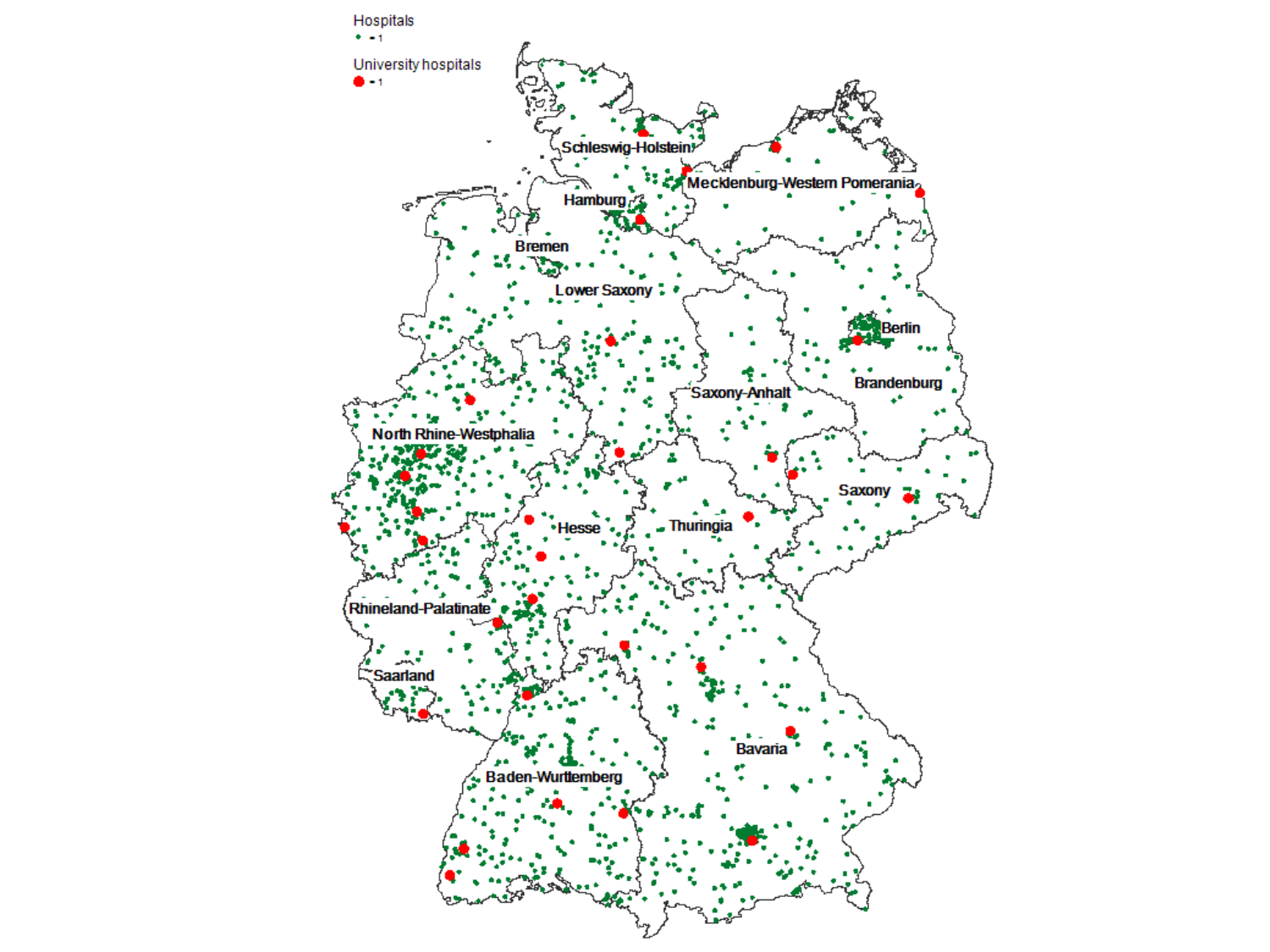

Supplement: Supplementary file 3 — Additional file 3. Hospitals and university hospitals in Germany per district, 2017. Dots are randomly placed within one district. * According to the directory of hospitals, there were 1,776 general hospitals and 35 university hospitals in Germany in 2017 with a total of 479,893 general hospital beds and 45,156 university hospital beds. A total of 19,442,810 patients were treated in hospitals what accounted for 141,152*1,000 patient-days and patients stayed in hospital for a mean of 7.3 days (range: 6.9-7.9 days by federal state). [file 13756_2021_1045_MOESM3_ESM.pptx]
